# *Ceratonia siliqua* pods (Carob) methanol extract alleviates doxorubicin-induced nephrotoxicity via antioxidant, anti-inflammatory and anti-apoptotic pathways in rats

**DOI:** 10.1007/s11356-023-28146-z

**Published:** 2023-06-21

**Authors:** Attia H. Atta, Shimaa A. Atta, Marwa S. Khattab, Tamer H. Abd El-Aziz, Samar M. Mouneir, Marwa A. Ibrahim, Soad M. Nasr, Shimaa R. Emam

**Affiliations:** 1 Pharmacology Department, Faculty of Veterinary Medicine, Cairo University, Giza, 1211 Egypt; 2 Immunology Department, Theodor Belharz Research Institute, Giza, 12411 Egypt; 3 Pathology Department, Faculty of Veterinary Medicine, Cairo University, Giza, 1211 Egypt; 4 Department of Parasitology and Animal Diseases, National Research Centre, 33 Bohouth St, DokkiGiza, 12622 Egypt; 5 Biochemistry and Molecular Biology Department, Faculty of Veterinary Medicine, Cairo University, Giza, 1211 Egypt

**Keywords:** *Ceratonia siliqua*, Carob, Doxorubicin, Nephrotoxicity, Antioxidant, Anti-Inflammatory, Anti-apoptotic, Phytochemistry

## Abstract

Doxorubicin (DOX) is an anti-neoplastic therapy, but its use is limited by its deleterious toxic effects including nephrotoxicity and cardiotoxicity. This work aimed at assessing the potential protective effect of *Ceratonia siliqua* methanol extract (CME) on DOX-induced nephrotoxicity in 5 groups of Wistar rats. Nephrotoxicity was induced experimentally by intraperitoneal (IP) injection of DOX (15 mg/kg). DOX increased serum creatinine, urea, sodium, and potassium levels. It elevated MDA levels in the renal tissue but decreased the concentration of GSH and the activity of GST, CAT, and SOD. Meanwhile, it decreased the level of immunomodulatory anti-inflammatory mediators: IL-10 and TGF-β, as well as the activity of MPO but increased the level of IL-6, TNF-α, and caspase-3 in the renal tissue. DOX has upregulated COX-2, caspase-9, and Bax gene expression and downregulated the Bcl-2 gene expression. Immunolabeling of renal tubular epithelium in DOX-intoxicated rats was moderate to strong against Bax, COX-2, and NF-kβ and weak against Bcl-2. Treatment with CME significantly restored the levels of kidney function parameters and the levels of oxidative stress markers. It stimulated the production of IL-10 and TGF-β and decreased the level of IL-6 and TNF-α. CME reverted the gene expression of COX-2, caspase-9, and Bax. Microscopically, CME alleviated the DOX-induced renal damage. Phytochemical analysis revealed the presence of 26 compounds in the CME. No signs of acute toxicity were recorded by CME up to 4000 mg/kg b. wt. orally into mice. Finally, CME could effectively alleviate the deleterious effects of DOX on the kidney. The safety of carob extract encourages its use in the preparation of valuable therapeutic agents.

## Introduction

The kidney is a vital organ in the body performing many essential functions including the regulation of the extracellular environment to maintain homeostasis, elimination of noxious metabolites, and detoxification drugs (Ferguson et al. 2008). The kidney is a major target organ for exogenous toxicants and endogenous toxic products. Nephrotoxicity is a renal-specific feature in which excretion does not go smoothly because of toxic metabolites or drugs (Finn and Porter 2003). Approximately 20% of nephrotoxicity is induced by drugs, but medication for aged persons increases the incidence of nephrotoxicity by up to 66% (Kim and Moon 2012). Anticancer drugs are well known for their nephrotoxic effect (Naughton 2008; Nagai and Takano 2010). Several mechanisms are reported to cause nephrotoxicity. These factors include inflammation, changes in glomerular hemodynamics, crystal nephropathy, tubular cell toxicity, and thrombotic microangiopathy (Schnellmann and Kelly 1999; Ferguson et al. 2008; Finn and Porter 2003).

Doxorubicin is a very effective therapeutic agent used for the management of different types of malignancies (Xiao et al. 2012). However, its use is associated with many toxic effects such as nephrotoxicity (El-Sheikh et al. 2012) and cardiotoxicity (Reis-Mendes et al. 2021). Doxorubicin has been administered for the treatment of various types of tumors in frequent (weekly) low doses (Chlebowski et al. 1984), single high doses (Benjamin et al. 1977), and as a continuous infusion (Garnick et al. 1983) depending on the pharmacokinetics and biological properties of doxorubicin, the response rate, the duration, and the toxicity (Jones et al. 1987). Moreover, two major signaling pathways explain DOX-induced apoptosis, the mitochondrial pathway and the death receptor pathway (Ryter et al. 2007). The mitochondrial pathway was reported t be the major signaling pathway that is involved in DOX-induced toxicities (Xiao et al. 2012). Mitochondrial cytochrome c is released into the cytoplasm as a result of mitochondrial dysfunction, in addition to the formation of a complex of cytochrome c, Apaf-1, and caspase-9 enzyme which is called apoptosome (Green and Reed 1998) that are the sequences of DOX induced-apoptosis through a mitochondrial pathway. Apoptosome activates caspase-3 which is a protease that leads to cell death (Beere et al. 2000). In addition DOX plays an important role in cellular toxicity (Arola et al. 2000) through apoptotic pathway activation that mediate DOX-induced oxidative stress to various biological macromolecules and membrane lipid peroxidation by reactive oxygen species (ROS) produced by redox cycling (Sun et al. 2016). Doxorubicin could induce mediators of fibrosis, inflammation, and oxidative/nitrative stress in the progression of renal injury initiated by glomerular podocyte damage (Szalay et al. 2015). Moreover, DOX stimulates the release of various pro-inflammatory mediators such as cyclooxygenase-2 (COX-2) (Abd El-Aziz et al. 2012) that are mediated mainly by upregulating the expression of the nuclear factor kappa-B (NF-κβ). It was reported that this inflammatory pathway plays an essential role in DOX-induced nephrotoxicity (Rashid et al. 2013).

*Ceratonia siliqua* fruit (carob) is used in the food industry as a flavoring, thickener, and stabilizer in food (Dakia et al. 2007). Carob pulp (pods) is the most important edible part of the carob fruit. Recently, it has been reported that carob induces a variety of pharmacological actions including antimicrobial, anti-inflammatory, anti-diarrheal, anti-ulcer, antioxidant, gastroprotective, and anti-constipation effects (Rtibi et al. 2015 and 2017). It has ant-diabetic (Qasem et al. 2018), hepatoprotective (Souli et al. 2015; Martić et al. 2022), and antioxidant and cytotoxic activities effects (Custodio et al. 2011; Ayache et al. 2020). It has also been found to be effective in neurodegenerative diseases (Lakkab et al. 2018) and has a protective effect on colon adenoma cells from the genotoxic impact of H2O2 (Klenow et al. 2009). *Ceratonia siliqua* contains a large number of phytocomponents in all parts such as phenolics, tannins, flavonoids, anthocyanins, glycosides, proteins, alkaloids, and minerals (Goulas and Georgiou 2019). The concentration of phenolic constituents in carob fruit, which is mainly phenolic acids and tannins (Stavrou et al. 2018), depends on genomic and environmental factors (Goulas et al. 2016) and the type of the solvent used for extraction (Goulas and Georgiou 2019). There is no study concerned with the evaluation of the protective effect of *Ceratonia siliqua* methanol extract (CME) on DOX-induced nephrotoxicity. The objective of this work was to assess the protective effect of CME on DOX-induced nephrotoxicity and to elucidate its mechanism of action by testing the associated antioxidant, anti-inflammatory, immunomodulatory, and apoptotic effects in the rat model.

## Material and methods

### Plant material

*Ceratonia siliqua* pods (carob) were purchased from a local herbal market and were identified by the Staff Members of the Department of Flora, Ministry of Agriculture, Giza, Egypt. A voucher sample was kept in the Pharmacology Department, Faculty of Veterinary Medicine, Cairo University. The seeds of the dried pods were removed, and the carob pulps were powdered in an electric blender. Two hundred grams of the dried powder were extracted with methanol 95% for 24 h, followed by percolation 5–7 times till complete extraction. The extract was filtered using Whatman filter No. 40. The methanol extract was concentrated under reduced pressure at a temperature not more than 50 °C using a Rotary evaporator (Heidolph 2000, Germany). The concentrated pasty extract was then dried and reserved at − 4 °C until subsequent use. The extract was freshly suspended in sterile phosphate buffer saline (pH 7.2) to a final concentration of 200 mg/ml. The methanol extract was used since it has the highest phenolic content (El Hajaji et al. 2011) which showed very good antioxidant and anti-inflammatory properties (Lachkar et al. 2016).

### Acute toxicity testing

Twenty mice were allocated randomly into 4 groups of 5 mice each. Before testing, the animals were fasted for 12 h (Atta and El-Sooud 2004) but allowed free access to drinking water. Rats in groups A, B, C, and D were orally administered 500, 1000, 2000, and 4000 mg/kg b. wt. of the carob pulp methanol extract, respectively. Mortality and symptoms of toxicity such as jerks and writhing were observed over 24 h and daily for up to 5 days.

### Phytochemistry

Gas chromatography (Agilent Technologies 7890A) interfaced with a mass selective detector (Agilent 7000 Triple Quad) and Agilent HP-5 ms capillary column (30 m × 0.25 mm ID and 0.25 μm film thickness) was used. The injector and the detector temperatures were adjusted to 200 °C and 250 °C, respectively. The flow rate was 1 ml/min. The acquisition mass range was 50–600. The recorded formulae of the components were identified by comparing their mass spectra and RT with those of NIST and WILEY library.

### Animals, treatments, and sampling

**Fig. 1:**
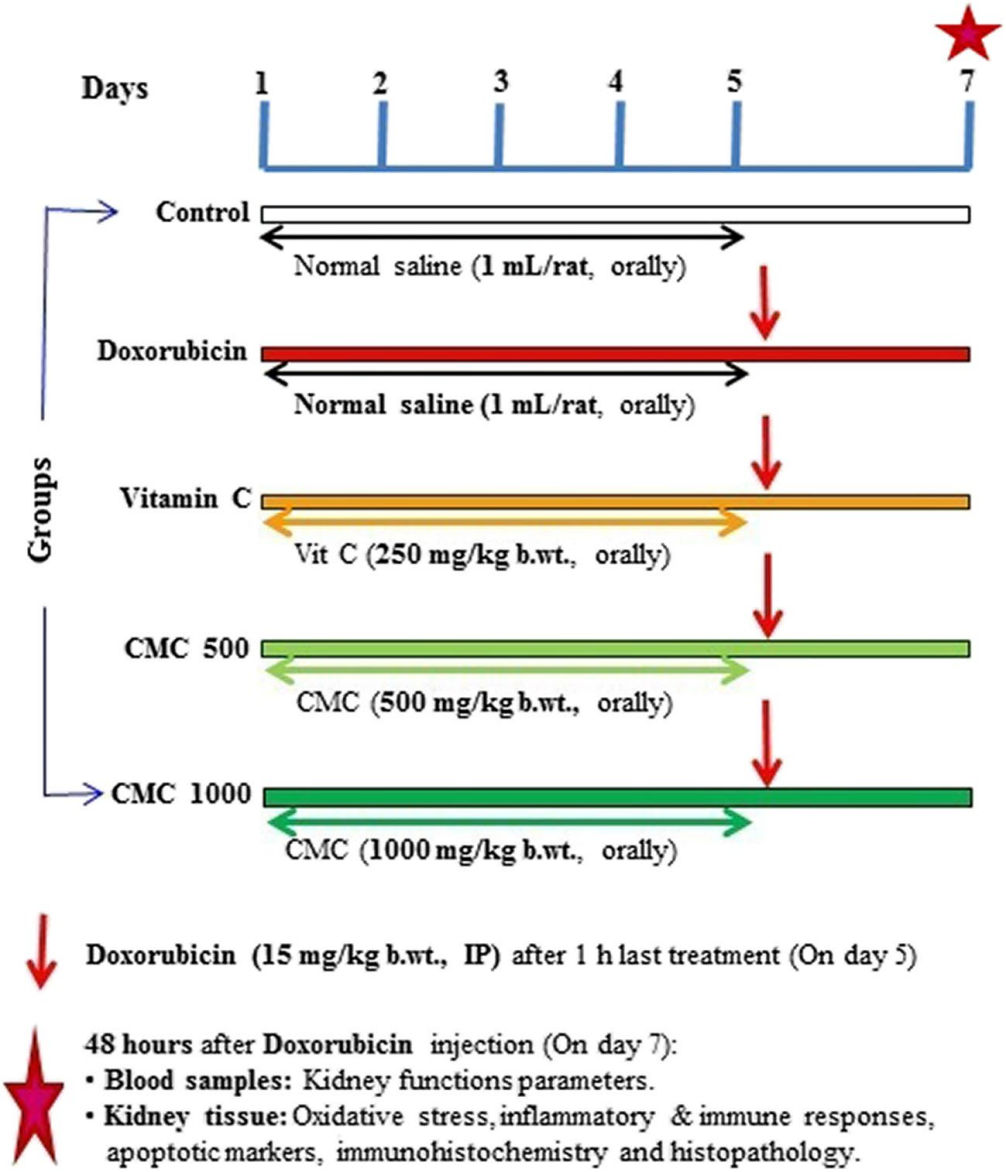
Experimental groups, treatments and sampling. CME500 and CME1000: rats treated with carob pod methanol extract at a dose of 500 and 1000 mg/kg respectively

Thirty-five Wistar rats of 200–250 g body weight were purchased from Animal Breeding House, National Research Centre, Giza, Egypt. Animals were reared under strict hygienic conditions for 7 days for acclimatization. Animals were randomly allocated into 5 equal groups. Rats of the first (control) and second group (Doxorubicin) were given normal saline (1 ml/rat) orally by gastric gavage for 5 days. Rats of the third group (vitamin C) were given vitamin C (a reference antioxidant) at a dose of 250 mg/kg b. wt. orally. Rats in the fourth (CME500) and fifth (CME1000) groups were given CME at a dose of 500 and 1000 mg/kg b. wt. respectively by the same route for the same period. The methanolic carob extract was used at a wide range of doses for a wide range of durations (Rtibi et al. 2015; Altınkaynak et al. 2018; Soleimanzadeh et al. 2020). Moreover, the anti-nociceptive and anti-inflammatory effects appeared after short periods (Alqudah et al. 2022; Lachkar et al. 2016). Therefore, the used doses of the methanolic carob extract were selected to represent a low dose of 500 mg kg^−1^ (group CME500) and a high dose of 1000 mg kg^−1^ (group CME1000) given for 5 days according to the previous studies (Rtibi et al. 2015; Qasem et al. 2018). On the fifth day, rats of groups 2–5 were given DOX at a dose of 15 mg/kg IP, 1 h after the last treatment dose (Ibrahim et al. 2019). The used doxorubicin dose was selected because it has been reported that administration of a heavy single dose of DOX caused serious toxic effects on kidney tissue (Speth et al. 1988). Moreover, experimental nephrotoxicity can be induced by intraperitoneal (IP) injection of doxorubicin at such heavy dose (11.5 to 20 mg/kg) (Wu et al. 2021; Ibrahim et al. 2019; Altınkaynak et al. 2018). After 48 h, samples of blood were taken from the retro-orbital plexus of veins of each animal in all groups under light anesthesia. Blood samples were left to clot to obtain clear serum after centrifugation (4000 rpm for 10 min). Experimental groups, treatments, and sampling are demonstrated in Fig. 1. Serum was preserved at − 20 °C for further estimation of kidney function parameters (creatinine, urea, calcium, potassium, sodium, and chloride). Rats were then euthanized with pentobarbital sodium (150 mg/kg b. wt. IP). Both kidneys were removed from all rats, washed, and weighed. A portion of the kidney tissue of each rat was weighed and washed in ice-cold saline (0.9%) and was used for the preparation of kidney tissue homogenate. Samples were homogenized with 9 volumes of ice-cold 1.15% solution of potassium chloride in 50 mmol/l potassium phosphate buffer solution (pH 7.4) using a homogenizer (Witeg, Germany). The homogenate 10% (w/v) was centrifuged at 4,000 rpm for 10 min at 4 °C using a cooling centrifuge (Sigma 3-18KS, Germany). The supernatant was kept at − 80 °C till used for assessment of oxidative stress, inflammatory/anti-inflammatory, immune responses, and apoptotic markers within a week. Specimens of the kidney were fixed in 10% neutral buffered formalin and used for Immunohistochemistry and histopathology. The remaining kidney tissue sample was kept at − 80 °C and used for assessment of gene expression using real-time PCR.

### Assessment of nephrotoxicity indices in serum

Serum creatinine (Houot 1985) and blood urea (Patton and Crouch 1977) levels were measured in the serum using kits purchased from Erba, Germany. Serum electrolytes; sodium, and potassium (Henary 1964), chloride (Schoenfeld and Lewellen 1964), and calcium (Gindler and King 1972) levels were determined using kits purchased from Centronic, Germany.

### Assessment of oxidative stress markers in kidney homogenate

Reduced glutathione (GSH) concentration using enzymatic recycling method (Rahman et al. 2006), the activities of superoxide dismutase (SOD) (Marklund and Marklund 1974), glutathione-S-transferase (GST) (Habig et al. 1974), and the lipid peroxide by-product as malondialdehyde (MDA) contents (Ohkawa et al. 1979) were estimated in the supernatant of kidney homogenate. All analytical chemicals were purchased from Sigma Aldrich, USA. The concentration of MDA was estimated using a commercial test kit obtained from Biodiagnostic Co, Egypt. These indices parameters were estimated using a spectrophotometer (T80 UV/VIS PG instrument Ltd, UK), catalase (CAT), and myeloperoxidase (MPO) activities were assessed using Sandwich-ELISA kit (SunLong Biotech Co, LTD, China). ELISA plates have been pre-coated with an antibody specific to CAT and MPO, according to the manufacturer’s instructions.

### Assessment of inflammatory/anti-inflammatory, immune response, and apoptotic markers in kidney tissue homogenate

The pro-inflammatory cytokines the interleukin-6 (IL-6) were measured by using Sandwich-ELISA rat kits (SunLong Biotech Co, LTD, China), as described in the manufacturer’s instructions. Tumor necrosis factor alpha (TNF-α) was measured by using a Sandwich-ELISA rat kit (Cloud-Clone Corp, China). ELISA plates have been pre-coated with specific antibodies to IL-6 or TNF-α, following the manufacturer’s instructions. The levels of the anti-inflammatory immunomodulatory mediators interleukin-10 (IL-10) and transforming growth factor-β (TGF-β) were also determined by Sandwich-ELISA rat kits (SunLong Biotech Co, LTD, China). ELISA plates have been pre-coated with an antibody specific to IL-10 and TGF-β, according to the manufacturer’s instructions. The apoptotic marker caspase-3 (Cas-3) was assessed by Sandwich-ELISA rat kit (SUNRED Co., China). ELISA plates have been pre-coated with an antibody specific to Cas-3. In brief, kidney tissue homogenates were incubated with the immobilized specific antibodies for each marker and visualized using horseradish peroxidase (HRP)-Tetramethylbenzidine (HRP-TMB) reaction.

### Immunohistochemistry and histopathology

B-cell lymphoma-2 protein-associated X protein (Bax), B-cell lymphoma-2 protein (Bcl-2), cyclooxygenase-2 (COX-2), and nuclear factor kappa (NF-κβ) were immunohistochemically stained in paraffin-embedded tissue sections after antigen retrieval using citrate buffer (pH 6). Primary antibodies against Bax (InVitrogen PA5-78,857, USA), Bcl-2 (InVitrogen PA5-27,094, USA), COX-2 (InVitrogen PA1-37,505, USA), and NF-κβ P65 (sc-8008, Santa Cruz Biotechnology, Inc., Santa Cruz, CA, USA) were applied to the slides, followed by endogenous peroxidase blocking by hydrogen peroxide. Secondary horseradish peroxidase (HRP)-labeled antibody was applied according to the protocol of the manufacturer (Universal PolyHRP DAB kit for mouse and rabbit, Genemed, Sakura Torrance, CA, USA). Secondary antibodies were used without primary antibodies in the negative control slides. The immunohistochemical (IHC) technique was followed as described by Magaki et al. (2019). Fixed kidney tissue was dried out in ascending concentrations of ethanol, cleared in xylene, and then embedded in paraffin. The tissue was sectioned by microtome (Lieca 2135, Germany) into 4 µm thickness sections and stained by hematoxylin and eosin stain. A light microscope (Olympus BX43, Japan) equipped with a digital camera (Olympus DP27 camera) was used for examination (Suvarna et al. 2019).

### Assessment of gene expression

The transcript level of COX-2, Cas-9, Bax, and Bcl-2 was assessed by the R-T PCR. The EasyRNA™ Cell/Tissue RNA Mini Kit (Biovision #K1337) was used for the extraction of the total RNA of the kidney tissue. The synthesis of the first-strand cDNA was carried out using SuperScript Reverse Transcriptases (Thermoscientific) according to the instructions of the manufacturer. Quantitative PCR was performed using Power Track™ SYBR Green Master Mix Applied Biosystems™ on an ABI Prism Step OnePlus Real-Time PCR System (Applied Biosystems) according to the manufacturer’s instructions. The primer sets used to amplify the target genes were designed using the primer 3 software (https://primer3.ut.ee/) using the Rattus norvegicus sequences in the GenBank. The primer sets of the assessed genes are listed in Table 1. The relative mRNA expression of the target genes was calculated as a fold change of the normal control after normalization to act as a reference transcript, using 2 − ΔΔCT methods.

**Table 1:** The primer sets of the assessed genes

Gene	Forward primer	Reverse primer	Product	Accession No
*cas-9*	CTGTGTTCCAAGGTCTCGGC	CCAGGCTCACTTAGCAAGGAA	149	NM_031632.2
*bax*	CACGTCTGCGGGGAGTCAC	TTCTTGGTGGATGCGTCCTG	248	NM_017059.2
*Bcl-2*	TCGCGACTTTGCAGAGATGT	CAATCCTCCCCCAGTTCACC	116	NM_016993.2
*cox-2*	CTCAGCCATGCAGCAAATCC	GGGTGGGCTTCAGCAGTAAT	172	NM_017232.3
*actb*	CCGCGAGTACAACCTTCTTG	CAGTTGGTGACAATGCCGTG	297	NM_031144.3

### Statistics

The mean ± SD of the obtained data in each group was calculated. The one-way analysis of variance (ANOVA) followed by Duncan’s multiple range test was applied to test the significance of differences between means of different groups. The significance was set at *P* < 0.05 (IBM SPSS Statistics 24.0).

## Results

### Acute toxicity

No symptoms of illness or discomfort appeared for a 5-day observation period in mice receiving graded doses of *Ceratonia siliqua* methanol extract up to 4000 mg/kg b. wt. administered orally. Thus, the LD50 value by oral route cannot be determined as no mortality was observed.

### Effect of CME on serum kidney function parameters

Serum creatinine, urea, sodium, and potassium concentrations were significantly (*P* < 0.01) increased in DOX-intoxicated rats, while serum calcium levels decreased significantly as compared to normal control rats. Treatment of DOX-intoxicated rats treated with CME 500, or 1000 mg/kg b. wt.), as well as vitamin C, significantly (*P* < 0.01) decreased serum creatinine, urea, sodium, and potassium while serum calcium level markedly (*P* < 0.01) increased in comparison with the DOX-intoxicated group. However, chloride concentration was not affected (Table 2).

**Table 2:** Effect of carob methanol extract (CME) and vitamin C on kidney functions parameters in the serum of rats with doxorubicin-induced nephrotoxicity (mean ± SE, *n* = 6)

Groups	Creatinine (mg/dl)	Urea (mg/dl)	Sodium (mEq/l)	Potassium (mEq/l)	Chloride (mEq/l)	Calcium (mg/dl)
Control	0.27 ± 0.02^c^	38.69 ± 6.4^c^	134.06 ± 3.4^c^	4.38 ± 0.47^b^	100.66 ± 3.3	10.89 ± 0.2^a^
Doxorubicin	0.68 ± 0.13^a^	55.56 ± 7.0^a^	143.13 ± 2.8^a^	5.45 ± 0.78^a^	97.76 ± 3.8	9.87 ± 0.2^c^
Vitamin C (250 mg/kg b.wt.)	0.45 ± 0.06^b^	46.46 ± 7.2^b^	135.75 ± 3.1^bc^	4.54 ± 0.34^b^	98.10 ± 2.2	10.37 ± 0.1^b^
CME500 mg/kg b.wt.	0.52 ± 0.07^b^	46.69 ± 4.3^b^	136.54 ± 1.7^bc^	4.62 ± 0.25^b^	98.40 ± 1.3	10.42 ± 0.2^b^
CME1000 mg/kg b.wt.	0.53 ± 0.05^b^	47.10 ± 4.7^b^	138.89 ± 2.2^b^	4.92 ± 0.21^ab^	99.74 ± 1.8	10.57 ± 0.3^b^
*P-*value	*P *= 0.001	*P *= 0.002	*P *= 0.001	*P *= 0.004	*P *= 0.07	*P *= 0.01

Means with different superscripts in the same column are significantly different at *P* < 0.05

### Effect of CME on oxidative stress markers in kidney tissue homogenate

GSH and GST concentration and CAT and SOD activities were significantly (*P* < 0.05) decreased, and MDA was significantly increased in the kidney tissue homogenate of rats with DOX-induced nephrotoxicity. Treatment of DOX-intoxicated rats with CME 500, and 1000 mg/kg b. wt., as well as vitamin C, significantly (*P* < 0.05) restored the values of oxidative stress markers (GSH, CAT, SOD, and MDA) to normal levels (Table 3).

**Table 3:** Effect of carob methanol extract (CME) and vitamin C on oxidative stress markers in the kidney tissue homogenate of rats treated with doxorubicin-induced nephrotoxicity (mean ± SE, *n* = 6)

Groups	GSH (nMol/100 mg protein)	GST (nMol/min/mg protein)	Catalase (pg/ml)	SOD (U/ml tissue)	MDA (mMol/g tissue)
Control	3.00 ± 0.80^c^	0.32 ± 0.02^a^	83.33 ± 9.44^b^	73.80 ± 2.71^b^	8.48 ± 0.36^b^
Doxorubicin	0.70 ± 0.10^d^	0.26 ± 0.01^b^	45.00 ± 2.98^d^	66.60 ± 6.56^c^	10.18 ± 1.54^a^
Vitamin C (250 mg/kg b.wt.)	9.20 ± 1.10^a^	0.27 ± 0.042^b^	62.50 ± 1.77^c^	83.80 ± 1.60^b^	7.72 ± 0.33^b^
CME500 mg/kg b.wt.	7.80 ± 0.70^b^	0.32 ± 0.05^a^	91.50 ± 8.60^a^	79.20 ± 9.52^b^	7.87 ± 0.05^b^
CME1000 mg/kg b.wt.	8.90 ± 1.40^a^	0.25 ± 0.01^b^	60.00 ± 7.07^c^	90.80 ± 3.87^a^	6.55 ± 0.43^c^
*P-*value	*P *= 0.001	*P *= 0.001	*P *= 0.001	*P *= 0.001	*P *= 0.001

Means with different superscripts in the same column are significantly different at *P* < 0.05

*GSH* reduced glutathione, *GST* glutathione-s-transferase, *SOD* superoxide dismutase, *MDA* malondialdehyde

### Effect of CME on the inflammatory, immune responses, and apoptotic markers in the kidney tissue homogenate

**Fig. 2:**
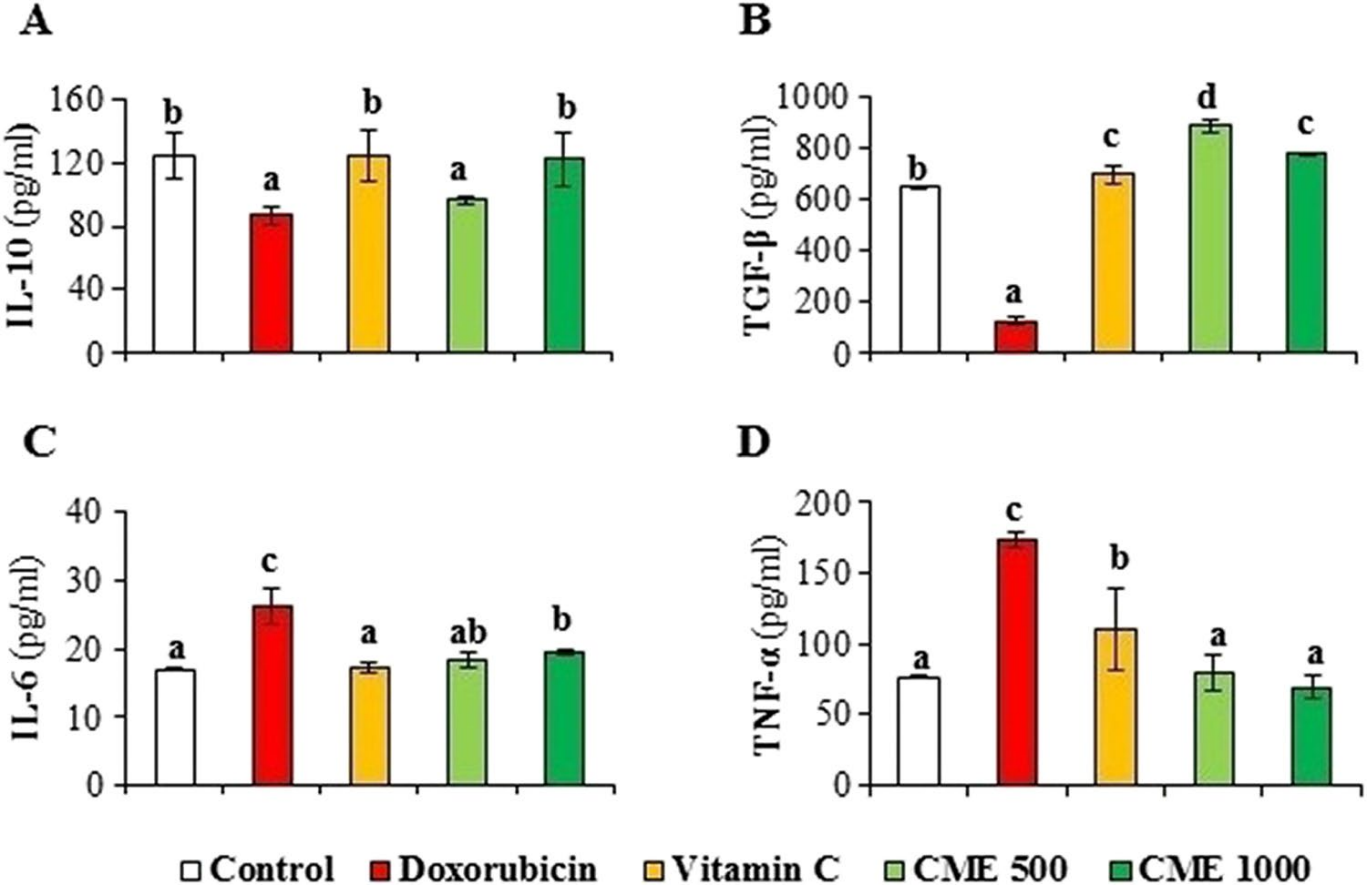
Effect of carob pod methanol extract and vitamin C on cytokine levels in kidney tissue homogenate of doxorubicin-induced nephrotoxicity in rats. (**A**) Interleukin-10 (IL-10), (**B**) transforming growth factor β (TGF-β), (**C**) interleukin-6 (IL-6), and (**D**) tumour necrosis factor alpha (TNF-α) (mean ± SD, *n* = 7). CME500 and CME1000: rats treated with carob pod methanol extract at a dose of 500 and 1000 mg/kg, respectively. Means with different superscripts in the same parameter are significantly different at *P* < 0.05

**Fig. 3:**
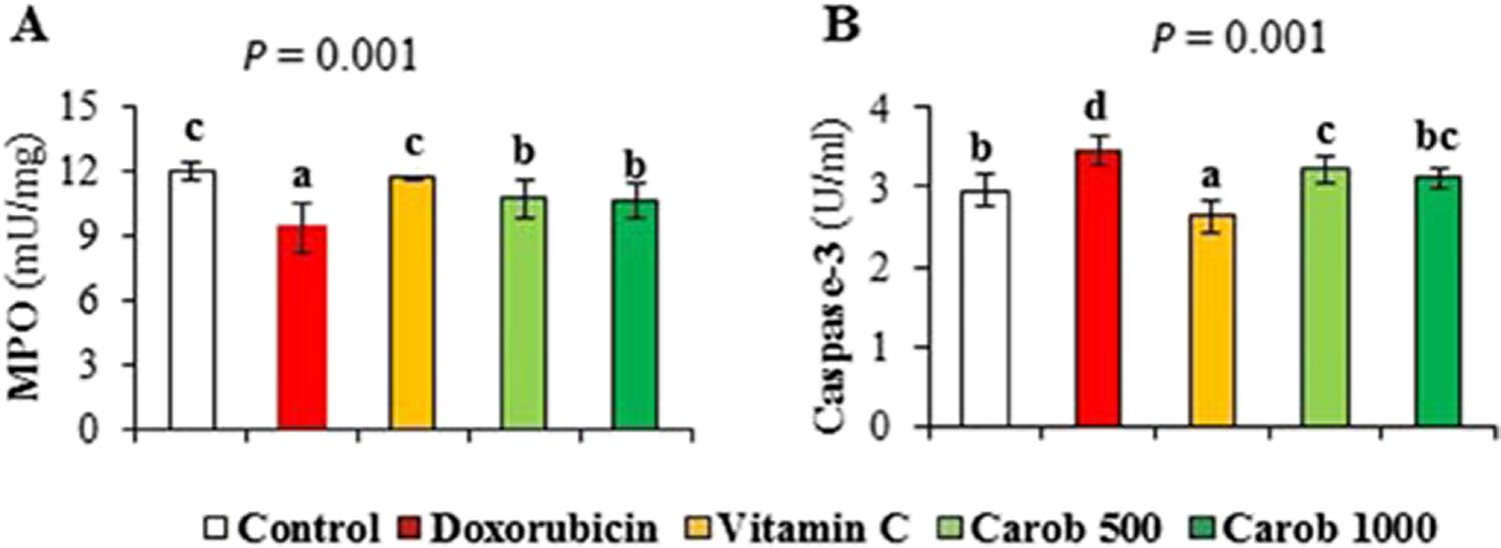
Effect of carob pod methanol extract and vitamin C on the level of (**A**) myeloperoxidase (MPO) and (**B**) caspase-3 in kidney tissue homogenate of doxorubicin-induced nephrotoxicity in rats (mean ± SD, *n* = 7). CME500 and CME1000: rats treated with carob pod methanol extract at a dose of 500 and 1000 mg/kg, respectively. Means with different superscripts in the same parameter are significantly different at *P* < 0.05

The level of the anti-inflammatory immunomodulatory cytokine, IL-10 was decreased in the kidney tissue homogenate of DOX-intoxicated rats (87.08 ± 6.21 pg/ml) as compared to the normal one (125.00 ± 14.14 pg/ml). Moreover, the anti-inflammatory transforming growth factor-β (TGF-β) level was also markedly decreased in the kidney tissue homogenate of the DOX-intoxicated group (105.83 ± 27.09 pg/ml) as compared to the normal one (646.66 ± 3.02 pg/ml). Treatment of DOX-induced nephrotoxic rats with vitamin C, low or high doses of the CME significantly, increased the IL-10 (Fig. 2A) and TGF-β levels (Fig. 2B). On the other hand, the pro-inflammatory markers IL-6 and the TNF-α were significantly increased in the DOX group (26.25 ± 2.62 pg/ml) as compared to normal one (17.16 ± 0.81 pg/ml). Treatment of DOX-intoxicated rats with vitamin C, low or high doses of CME significantly, decreased the levels of IL-6 (Fig. 2C) and the TNF-α (Fig. 2D) as compared to DOX-intoxicated one. MPO was decreased in the kidney tissue of the DOX group (9.32 ± 1.19 mU/mg) as compared to the normal control (11.98 ± 0.36 mU/mg). Administration of CME at the small (10.71 ± 0.86 mU/mg) or at the large dose (10.66 ± 0.81 mU/mg) restored significantly the level of MPO. Vitamin C (a standard drug) has normalized the level of MPO (Fig. 3A). On the other hand, the proapoptotic marker, Cas-3, was significantly increased in the renal tissue of DOX-nephrotoxic rats as compared to the normal level (3.47 ± 0.19 vs 2.96 ± 0.20 U/ml, respectively). Treatment of DOX-intoxicated rats with CME (low or high dose) as well as with vitamin C has decreased significantly the level of Cas-3 (Fig. 3B).

### Effect on the transcript level of COX-2, Cas-9, Bax, and Bcl-2

**Fig. 4:**
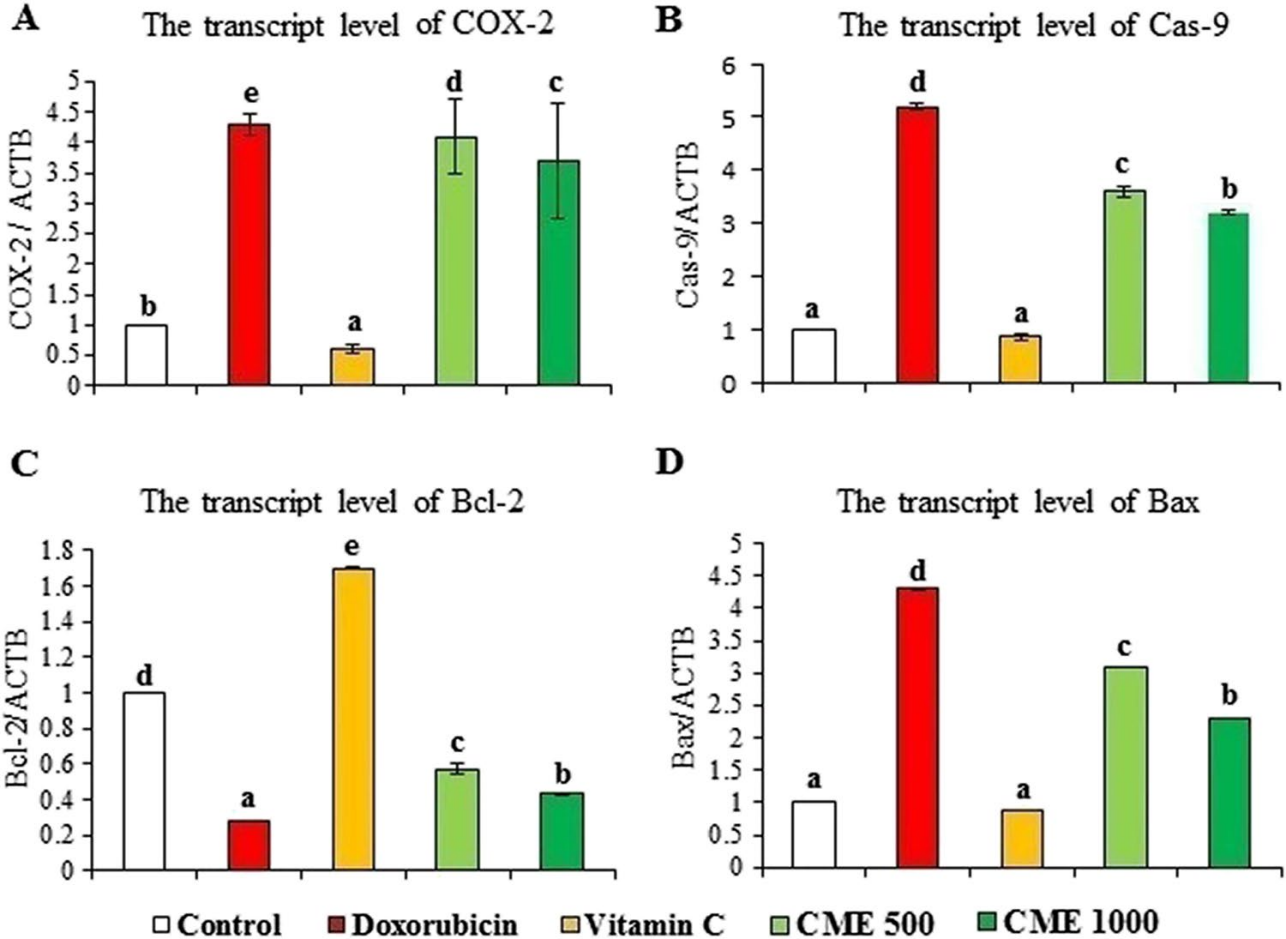
Effect of carob pod methanol extract and vitamin C on the transcript level of (**A**) COX-2, (**B**) Cas-9 (), (**C**) Bcl-2, and (**D**) Bax in the kidney tissue of rats with doxorubicin-induced nephrotoxicity (mean ± SD, *n* = 7). CME500 and CME1000: rats treated with carob pod methanol extract at a dose of 500 and 1000 mg/kg, respectively. COX-2, cyclooxigenase-2; Cas-9, caspase-9; Bax, B-cell lymphoma-2 protein-associated X protein; Bcl-2, B-cell lymphoma-2 protein. Means with different superscripts in the same parameter are significantly different at *P* < 0.05

The DOX-intoxicated group showed significant up-regulation of COX-2, Cas-9, and Bax transcripts as well as a significant downregulation of the anti-apoptotic marker, Bcl-2. Administration of CME alleviated the injurious effects induced by DOX as it suppressed the over-expression of COX-2, Cas-9, and Bax transcripts in a dose-dependent manner and significantly up-regulated the Bcl-2 gene (Fig. 4A–D).

### Immunohistochemistry of Bax, Bcl-2, COX-2, and NF-κβ

**Fig. 5:**
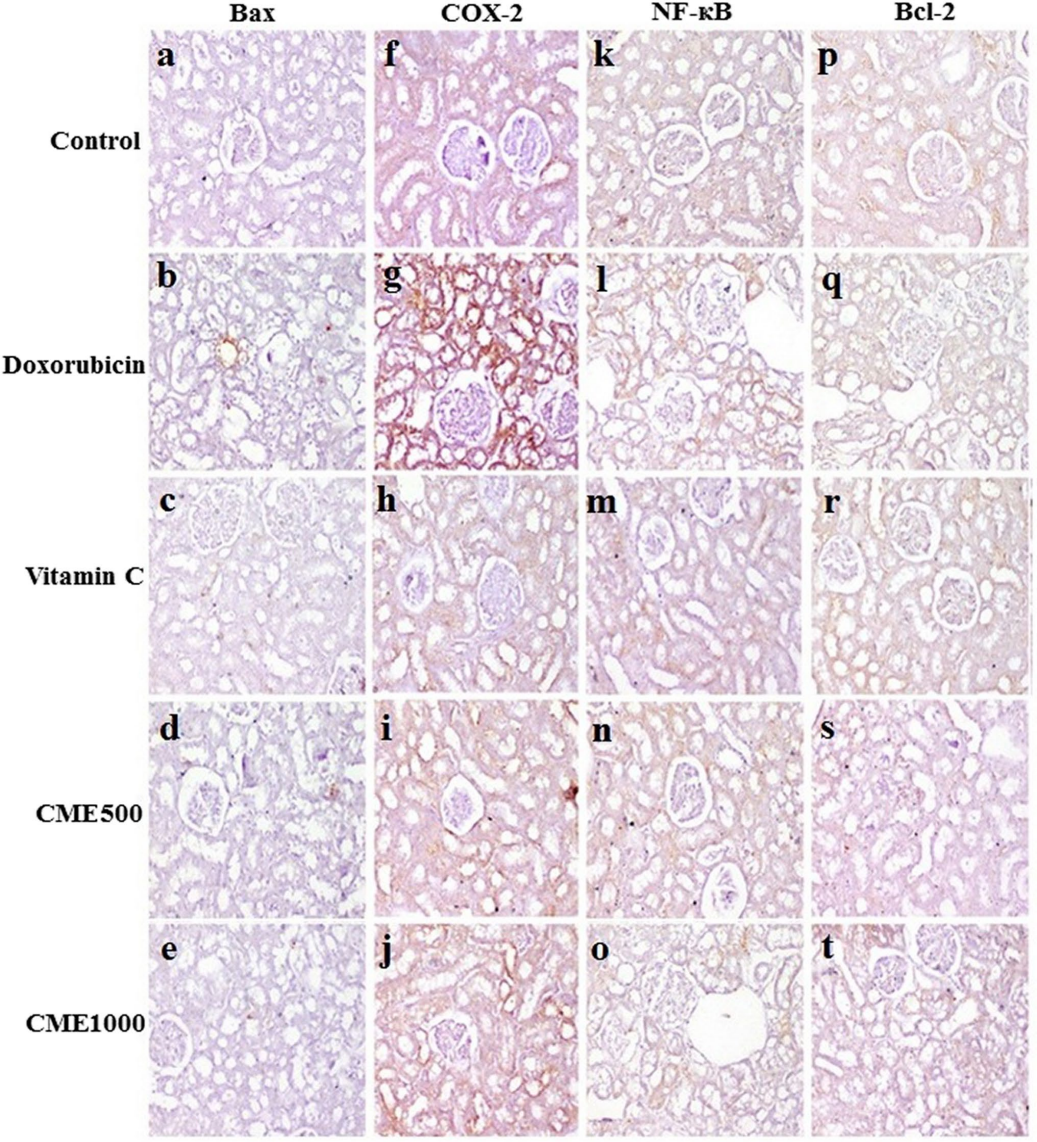
Immunohistochemistry of renal tubular epithelium in different groups showing; Bax immunolabeling which was weak in normal control (**a**), moderate in Doxorubicin-intoxicated rats (**b**), weak in vitamin C (**c**), carob methanol extract (CME)500 (**d**), and CME1000 (**e**) groups; COX-2 immunolabelling which was moderate in control group (f), strong in doxorubicin group (**g**), and weak in Vit. C (**h**), CME500 (**i**), and CME1000 (**j**) groups; NF-kB immunolabelling which was weak in the control group (**k**), moderate in the doxorubicin group (**l**), weak in Vit. C (**m**), CME500 (**n**), and CME1000 (**o**) groups; Bcl-2 immunolabelling which was moderate in control group (**p**), weak in doxorubicin group (**q**), moderate in Vitamin C (**r**), CME500 (**s**), and CME1000 (**t**) groups (immunoperoxidase and hematoxylin counterstain, X200). CME: carob pod methanol extract. CME500 and CME1000: rats treated with carob pod methanol extract at a dose of 500 and 1000 mg/kg, respectively. Bax, B-cell lymphoma-2 protein-associated X protein; COX-2, cyclooxigenase-2; NF-kβ, nuclear factor kappa; Bcl-2, B-cell lymphoma-2 protein

**Fig. 6:**
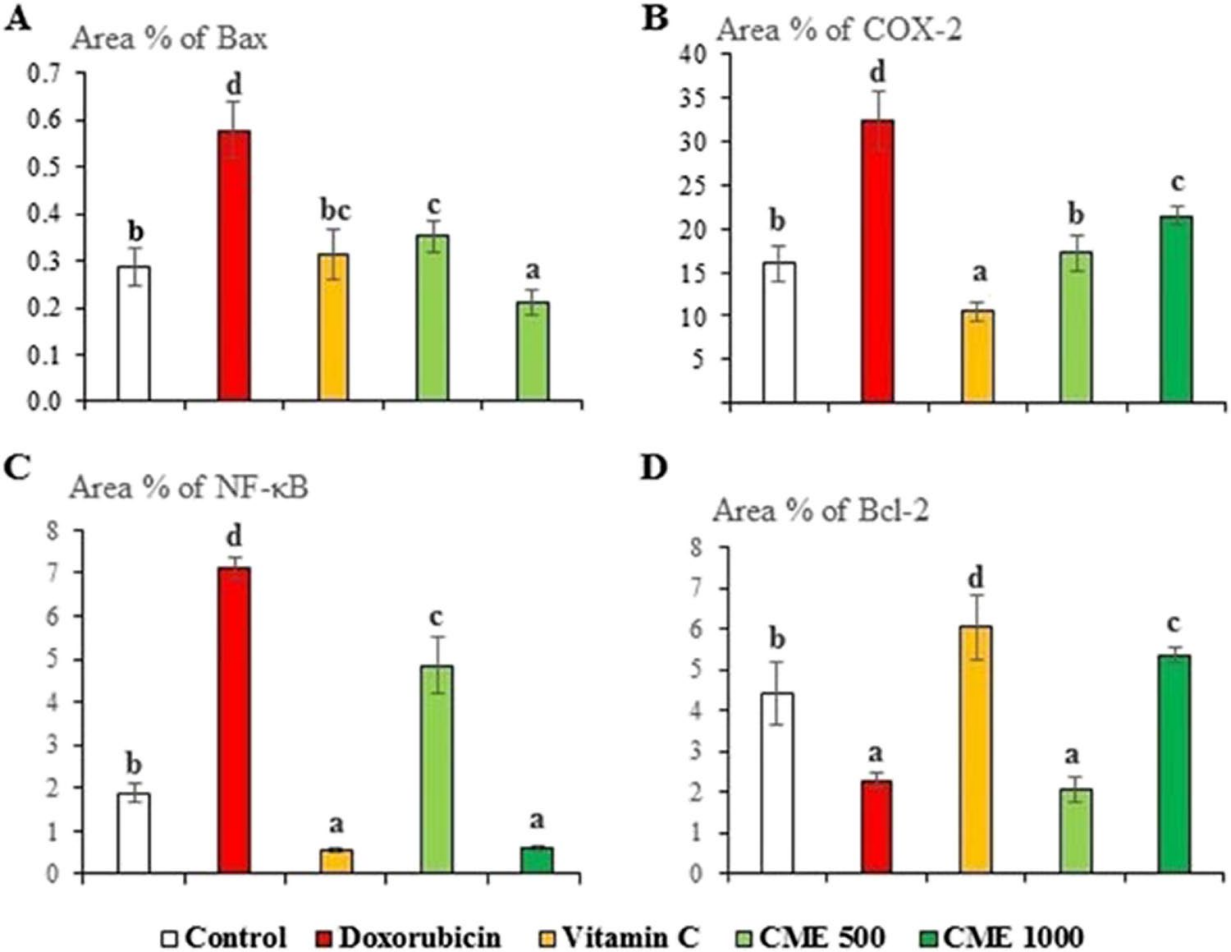
The area precents of (**A**) B-cell lymphoma-2 protein-associated X protein (Bax), (**B**) cyclooxigenase-2 (COX-2), (**C**) nuclear factor kappa (NF-kβ**)**, and (**D**) B-cell lymphoma-2 protein (Bcl-2) in different groups (mean ± SD, *n* = 7). CME500 and CME1000: rats treated with carob pod methanol extract at a dose of 500 and 1000 mg/kg, respectively. Means with different superscripts in the same parameter are significantly different at *P* < 0.05

Immunolabeling of renal tubular epithelium in control against Bax, COX-2, NF-kβ, and Bcl-2 was weak to moderate in control rats. Immunolabeling of tubular epithelium in DOX-intoxicated rats was moderate to strong against Bax, COX-2, NF-kβ, and Bcl-2. Immunolabeling of epithelium in DOX-intoxicated rats treated with vitamin C, CME 500, or 1000 mg kg^−1^ was mostly weak against Bax, COX-2, NF-kβ, and Bcl-2 (Fig. 5a–t). The area percent of Bax, Cox-2, and NF-κβ expression was markedly increased while the area percent of Bcl-2 was markedly decreased in DOX-nephrotoxic rats compared to normal ones. Treatment of DOX-intoxicated rats with CME at small and large doses as well as vitamin C has markedly decreased the area percent of Bax, Cox-2, and NF-κβ expression and increased the area percent of Bcl-2 expression (Fig. 6a–d).

### Histopathological findings

**Fig. 7:**
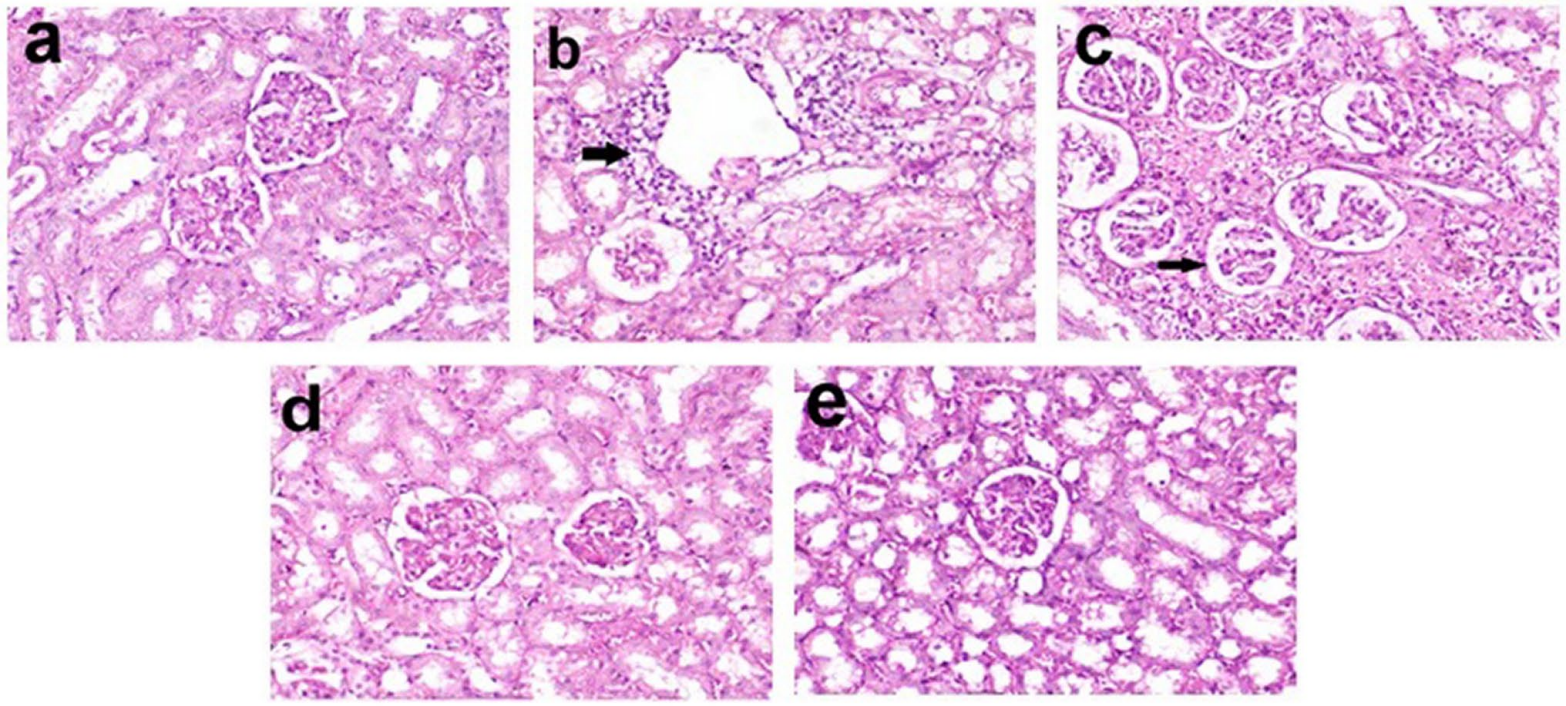
Histopathology of kidney from different groups: **a** normal histological structure of kidney in the normal control group; **b** glomerular atrophy, tubular dilatation, and degeneration with interstitial leukocyte infiltration (arrow) in doxorubicin-intoxicated group; **c** glomerular atrophy (arrow) and tubular degeneration in vitamin C-treated group; **d** moderate tubular degeneration in CME500, and **e** mild tubular degeneration in CME1000 (H&E, X200). CME500 and CME1000: rats treated with carob pod methanol extracts at a dose of 500 and 1000 mg/kg, respectively

Microscopical examination of renal tissue showed normal histological structure in control rats (Fig. 7a). In the DOX-intoxicated group, the glomeruli were atrophied and showed dilated Bowman’s capsules. Moreover, the renal tubules were dilated and the tubular epithelium was vacuolated and sometimes exfoliated in the lumen. Moderate multifocal mononuclear leukocyte infiltration was observed in the interstitial tissue (Fig. 7b). In DOX-intoxicated rats treated with vitamin C, the histopathological lesions were less severe compared to the non-treated group (Fig. 7c). The renal lesions were alleviated partially in CME500 (Fig. 6d) and were alleviated almost completely in CME1000 compared to DOX-intoxicated group (Fig. 7e).

### Phytochemical constituents

**Fig. 8:**
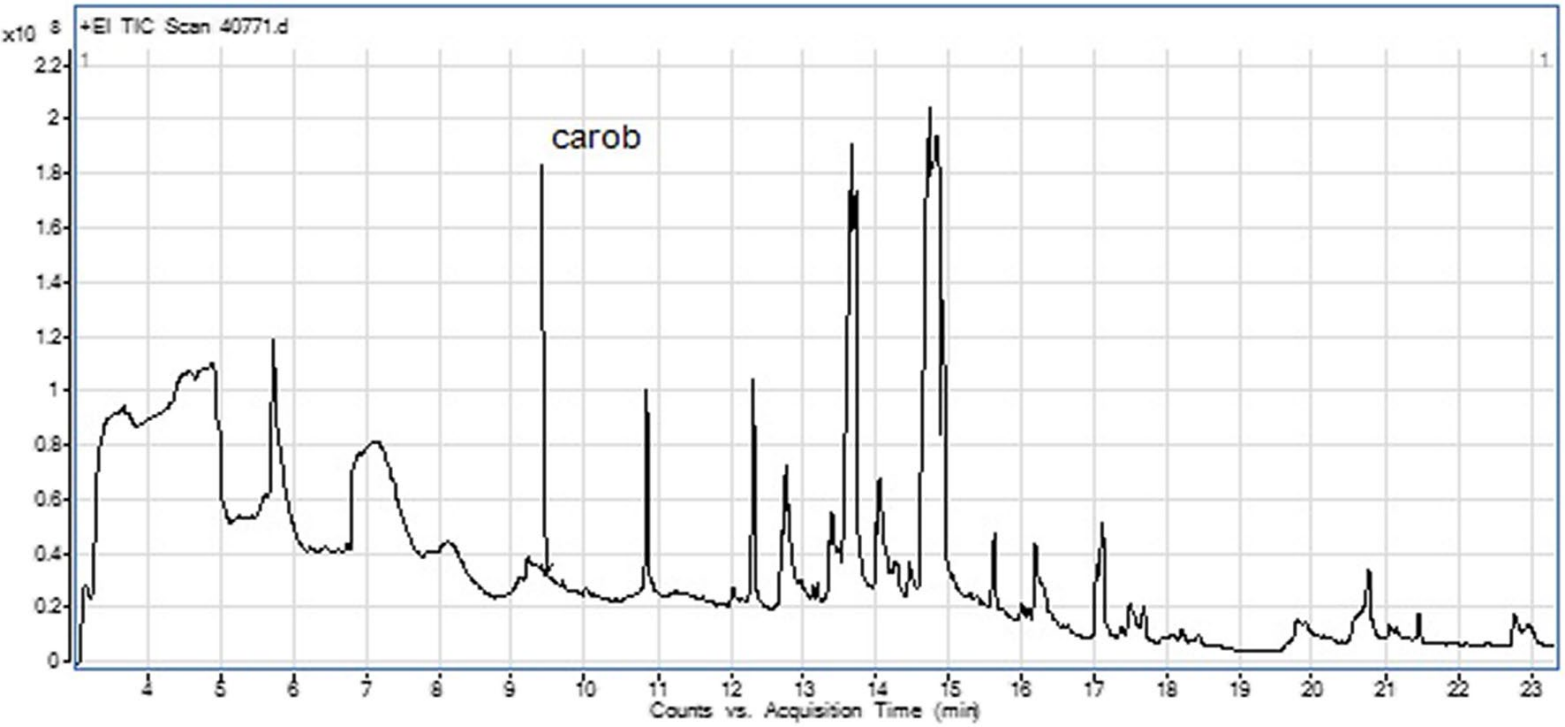
Gas chromatography/mass spectrometry (GC/MS) showing peaks of phytoconstituents of the methanol extract of carob pods

GC/MS analysis of the methanol extract of carob pods revealed the presence of 26 compounds (Fig. 8, Table 4). The major (over 5%) components were linolenic acid (28.86%), malic acid (15.28%), n-hexadecanoic acid (13.29%), and 2,3-butanediol (10.24%).

**Table 4:** 8 Gas chromatography/mass spectrometry (GC/MS) showing the phytoconstituents of the methanol extract of *Ceratonia siliqua* (carob pods)

No	RT (min)	Name	Area sum%
1	4.83	2,3-Butanediol	10.24
2	5.70	Butanoic acid, 2-ethyl-	4.73
3	7.13	Malic acid	15.28
4	9.27	Ascaridiol	1.43
5	10.30	Dodecanoic acid, ethyl ester	0.50
6	10.80	Dodecanoic acid, ethyl ester	2.53
7	12.06	Myristic acid	0.35
8	12.27	D-Gluconic acid, $\delta$-lactone	1.79
9	12.70	Pentadecanoic acid	2.99
10	13.13	*Flavonols 3',4',5,7-OH, 3-O araglucoside*	0.56
11	13.70	n-Hexadecanoic acid	13.29
12	14.04	Heptadecanoic acid	2.15
13	14.50	Silybin B	0.55
14	14.80	Linolenic acid	28.86
15	15.59	Eicosanoic acid	1.07
16	16.10	D-Pinitol	1.99
17	17.05	17-Octadecynoic acid	3.00
18	17.49	Octadecanedioic acid	0.72
19	17.62	2-Hexadecanol	0.29
20	18.20	Scutellarein tetramethyl ether	0.30
21	19.79	1-Heptatriacotanol	1.53
22	20.70	Quercetin 3-glucoside	2.63
23	21.06	Phythol	0.70
24	21.44	Thunbergol	0.82
25	22.75	$\delta$-Tocopherol	0.93
26	22.90	Colchifoleine	0.79

## Discussion

The results of the acute toxicity of *Ceratonia siliqua* (carob) showed that the carob methanol extract caused no symptoms of illness or discomfort indicating that the oral administration of the methanol extract in doses up to 4000 mg/kg was safe. The LD50 value could not be determined also by intraperitoneal route in mice as no lethality was observed up to 2000 mg/kg (Ben et al. (2020). It has been reported (Buck et al. 1976) that plant extracts with LD50 of more than 5000 mg/kg are considered nontoxic. Moreover, it has been reported that oral administration of the methanol extract of carob during 14-day experimental course in rats showed no symptoms of toxicity up to 5000 mg/kg (Qasem et al. 2018). Therefore, the present results suggest that the use of carob extract as a potential source for therapeutic agents is safe and without acute toxic effects.

Nephrotoxicity induced by drugs is a major health problem associated with severe renal tubular impairments and may lead to acute renal failure and a high rate of morbidity and mortality (Hussain et al. 2021). The rat model was successfully used to evaluate drug-induced amelioration of kidney function since their intra-renal enzyme distribution is similar to that in humans (Saad et al. 2009). DOX is a very effective chemotherapeutic agent and has useful applications for the treatment of several types of malignancies (Xiao et al. 2012; Zhu and Lin 2021); however, its use is limited by the deleterious effects on the kidney and various biological membranes (Sabapathy et al. 2021; Mahzari et al. 2021). In the present work, we aim to explore the potential preventive effect of the methanol extract of *Ceratonia siliqua* (carob) against doxorubicin-induced nephrotoxicity by testing its effects on kidney function, inflammatory/anti-inflammatory cytokines, oxidant/antioxidant markers, and apoptosis cascade and confirm our results by histopathological and immunohistochemical findings and by gene expression markers analysis.

In this study, further evidence of DOX-induced deleterious effects on renal function as indicated by elevation of serum creatinine, urea, serum sodium, potassium, and calcium levels is elucidated. The elevated levels of serum creatinine, urea, and electrolytes are general indices of kidney injury impelled by drug treatment (Hussain et al. 2021; Xing et al. 2022). The decreased activity of renal SOD, GST, and CAT accompanied by GSH depletion in the DOX-intoxicated rats is probably due to the increased generation of reactive free radicals (hydroxyl and superoxide radicals) (Nagai et al. 2018; Soltani Hekmat et al. 2021) and hence peroxidation of the lipid membrane and increased MDA production, the lipid peroxide by-product (Mahzari et al. 2021). The deleterious effects of DOX were also confirmed by histopathology and immunohistochemical analysis in the present results as well as in previous studies (Soltani Hekmat et al. 2021). Pre-treatment with CME has downregulated these parameters to a level comparable to control and maintained a normal histopathological picture. Similar protective effects of *Ceratonia siliqua* have been reported against 6-hydroxydopamine in Zebrafish (Abidar et al. 2020).

The enhanced production of ROS, decreased antioxidant enzymes in the kidney tissue altering the glomerular capillary permeability and inducing tubular degeneration, might be the underlying mechanism of DOX-induced renal damage (Carvalho et al. 2009; Sun et al. 2016) and the release of the cell cytokines including IL-1β, IL-6, and tumor necrosis factor alpha TNF-α, and NF-κβ. These cytokines play a central role in inflammation (Turner et al. 2014; Zhou et al. 2020) and trigger apoptotic cascades and their gene expression (Xiao et al. 2012; Wu et al. 2021). On the other hand, the nuclear factor E2-related factor 2 (Nrf2) is the main regulator of anti-oxidative responses (Vomund et al. 2017). Moreover, it has been shown that DOX caused downstream of the antioxidant genes and proteins, leading to the reduction of both mRNA and protein expressions of Nrf2 and hence intensifying the DOX-induced oxidative stress (Zhang et al. 2017; Kamble and Patil 2018). The observed increased generation of various inflammatory cytokines including COX-2, IL-6, and TNF-α as confirmed by upregulation of their gene expression triggers apoptotic cascades (Xiao et al. 2012; Wu et al. 2021). Moreover, the increased level of these pro-inflammatory mediators in sequence leads to more tissue injury and further stimulation of NF-κβ which amplifies inflammatory signals causing the production of more inflammatory cytokines including TNF-α) and interleukin-1β (IL-1β) and various chemokines which cause widespread inflammation and exacerbates tissue injury (Natarajan et al. 2018) which is considered a vital pathway in DOX-induced nephrotoxic effect (Rashid et al. 2013). Recently, a significant increase in the cardiorenal pro-inflammatory cytokines viz TNF-α and IL-6 in DOX-intoxicated rats was previously reported (Xing et al. 2022). The increased expression of the NF-κβ mediates the decreased level of the IL-10 and TGF-β in kidney tissue as has been reported in this study as well as others (Abd El-Aziz et al. 2012; Rashid et al. 2013). In DOX-treated rats, MPO was decreased as compared to normal rats. The decrease in MPO in DOX-treated animals may be due to early impairment of the enzyme leading to less subsequent oxidative stress (Reis-Mendes et al. 2021). The oxidative damage pathway has been thought to be a key anticancer mechanism of doxorubicin (Gupta and Srivastava 2012; Das et al. 2022). Treatment of DOX-intoxicated rats with CME has restored the pro-inflammatory cytokine (TNF-α, IL-6) level. IL-10 is an anti-inflammatory immunoregulatory cytokine whose primary function is to limit inflammatory responses (Bedke et al. 2019). Enhanced production of the anti-inflammatory cytokine (IL-10) mediates protection against several inflammatory conditions by preventing tissue damage (Kalkal et al. 2022; Short et al. 2022). IL-10 can inhibit the release of inflammatory cytokines such as IL-1, TNF-α, and IL-12 by the T cells and antigen-presenting cells (Cho et al. 2015; Brooks et al. 2010). It can also inhibit the production of chemokines, inhibit antigen presentation, and regulate immunoglobulin group switch in B cells (particularly to the IgG4 subclass) (Akdis et al. 1998). Moreover, IL-10 has clear immunoregulatory properties (Saraiva and O’Garra 2010; Couper et al. 2008) and enhances the transcription of genes associated with cell cycle progression and anti-apoptotic genes, such as Bcl (Sinuani et al. 2013). TGF-β is another cytokine whose functions are closely related to IL-10 (Li and Flavell 2008; Sinuani et al. 2013). TGF-β and IL-10 were identified as important immunomodulators anti-inflammatory that help to limit inflammation by controlling the development, maintenance, and activity of many immune cells (Drewry and Harty 2020). TGF-β regulates the differentiation, proliferation, hypertrophy, migration, and apoptosis of intraglomerular and tubular cells, regulates remodeling of the extracellular matrix, and promotes interstitial and glomerular fibrosis and the development of glomerulosclerosis (Böttinger 2007; Blobe et al. 2000). The infiltration of mononuclear inflammatory cells into the interstitium usually proceeds fibrosis. These cells secrete chemokines and cytokines that enhance the differentiation of the resident tubular epithelial cells into matrix-producing fibroblasts (Liu 2006; Fan et al. 1999; Kang et al. 2010). TGF-β and IL-10 may act synergistically to regulate the production of proinflammatory, chemokines cytokines, and nitric oxide by mononuclear cells. The decreased level of the IL-10 and TGF-β in the kidney of DOX-intoxicated rats indicates that DOX interfere with the establishment of adaptive immune responses. Similarly, it has been reported (Lubis et al. 2019) that doxorubicin suppressed the immune system in doxorubicin-treated rats as shown by suppression of phagocytosis, inhibition of lymphocyte proliferation, capacity and activity of macrophages, and downregulation of IL-10. Treatment of DOX-intoxicated rats with CME has enhanced the release of the anti-inflammatory and the immunomodulator cytokine IL-10 and the proteins TGF-β and inhibited the production of pro-inflammatory cytokine expression of TNF-α, IL1b, and IL-6 (Aboura et al. 2017), an effect that can counteract the DOX-induced stimulation of the production of the pro-inflammatory cytokines IL-6, COX-2, and TNF-α. The present results may be underlying the mechanism of the anti-inflammatory effects exhibited by *Ceratonia siliqua* leaves ethanol extracts (Alqudah et al. 2022).

Apoptosis is a process of controlled cell death in multicellular organisms, and its management is critical for normal growth, development, homeostasis, and cancer treatment. Changes in normal apoptosis can result in aberrant cell growth, excessive cell division, and mutation accumulation. Several molecular factors such as Bcl-2 and Bax play a crucial role in the implementation of apoptosis (Youle and Strasser 2008). Bax is an important initiator of mitochondrial-regulated cell death through its lethal activity of the outer membrane of the mitochondria. The physiological function of Bax is to ensure tissue homeostasis, and its dysregulation leads to abnormal cell death (Spitz and Gavathiotis 2021). Caspases are a large family of cysteine proteases that are essential for the initiation and execution of apoptosis (Fan et al. 2005; Sakamaki and Satou 2009). Apoptosis induced by DOX was evaluated by analysis of COX-2, Cas-9, Bax, and Bcl-2 gene expression as well as by immunohistochemical analysis of Bax, COX-2, NF-kβ, and Bcl2. DOX decreased anti-apoptotic Bcl-2 gene expression and upregulated both Cox-2, Bax, and Cas-9 genes. DOX-induced apoptosis is linked to the generation of ROS (Simon et al. 2000; Ozben 2007). Bcl-2 protein is a crucial regulator in the apoptotic pathway which triggers and accelerates cell death. DOX inhibits the expression of Bcl-2 antiapoptotic protein by forming Bcl-2/Bax heterodimers (Morgan et al. 2021). The caspase family is a group of cysteine proteases that trigger apoptosis. The activation of caspases leads to the induction of apoptotic pathways (Ebedy et al. 2022).

CME downregulated significantly the expression levels of the Bax, COX-2, and caspases, an effect which might be the underlying mechanism by which CME suppressed the DOX-induced nephrotoxicity. Bax, a member of Bcl-2 family proteins, undergoes conformational changes and becomes translocated to the mitochondria to initiate apoptosis when activated. In the present study, immunohistochemical studies as well as gene expression showed increased Bax positivity in renal tissues of DOX -intoxicated rats. This result is following other research which indicated that DOX mediates apoptosis through the activation of Bax (Mostafa et al. 2021). The DOX-intoxicated rats treated with vitamin C, CME 500, and CME 1000 mg/kg b.wt. decreased Bax expression as compared to the DOX-intoxicated group. On the other side, the decreased gene expression of Bcl-2 in the DOX group was similar to that previously reported by Vu et al. (2020) indicating induction of apoptosis. This detrimental effect of DOX was however relieved in the vitamin C and CME 1000 mg/kg -treated rats.

The NF-kβ is believed to be responsible significantly for controlling the transcription of various pro-inflammatory cytokines (Baldwin 1996). The results of this study confirm that DOX-induced nephrotoxicity is through stimulation of the NF-κβ signaling pathway (Wang et al. 2002; Mantawy et al. 2014) which was significantly decreased by vitamin C and CME treatment. The protective effect of CME could be attributed to its active constituents. One of the major constituents is linolenic acid (28.86%) which had presented cardioprotective and radioprotective effects (Poorani et al. 2020). Moreover, it decreased the levels of inflammatory cytokines and chemokines in an in vitro study** (**Morin et al. 2022). *Ceratonia siliqua* contains diverse bioactive phytoconstituents with high antioxidant activity such as phenolic compounds, flavonoids, alkaloids, and tannins (Lakkab et al. 2018). The antioxidant activity of *Ceratonia siliqua* has been reported to be strongly related to the high level of its phenolic compounds (Lachkar et al. 2016). In this study, methanol extract was used since it has the highest phenolic content (El Hajaji et al. 2011). Therefore, it could be suggested that the protective effect against lipid peroxidation caused by ROS in tissue and prevention of the depletion of the antioxidant enzyme; SOD, CAT, and GSH are attributed mostly to its phenolic content. In addition, *Ceratonia siliqua* aqueous pod extract was reported to protect the gastric mucosa via its anti-inflammatory and antioxidant activities maintaining a normal macroscopic and histological picture (Lachkar et al. 2016). Plant flavonoids have also been reported to alleviate gingival inflammation via the suppression of nuclear NF-κβ translocation and myeloperoxidase activity (Gugliandolo et al. 2019). The reported α-linolenic acid as a major component is an omega-3-fatty acid known to induce anti-inflammatory activity (Otuechere and Farombi 2020) which may explain the reported inhibition of the pro-inflammatory mediators.

## Conclusions

In a rat model of DOX-induced nephrotoxicity, the methanol extract of carob showed dose-dependent positive impacts with promising anti-inflammatory, antioxidant, and antiapoptotic activities as evidenced also by preserving the histopathological features of the kidney tissue. Further in-depth studies are required to explore the bioactive constituents in the methanol extract of carob pods aiming at the preparation of useful therapeutic agents.

## Data Availability

The data used in the present study are available from the corresponding author on reasonable request.

## Abbreviations

Bax: B-cell lymphoma-2 protein-associated X protein
Bcl-2: B-cell lymphoma-2 protein
Cas-3: Caspase-3
Cas-9: Caspase-9
CAT: Catalase
CME: *Ceratonia siliqua* Methanol extract
COX-2: Cyclooxigenase-2
DOX: Doxorubicin
ELISA: Enzyme-linked immunosorbent assay
GC/MS: Gas chromatography/mass spectrometry
GSH: Reduced glutathione
GST: Glutathione-s-transferase
IL-6: Interleukin-6
IL-10: Interleukin-10
IP: Intraperitoneal
MDA: Malondialdehyde
MPO: Myeloperoxidase
NF-κβ: Nuclear factor kappa beta
ROS: Reactive oxygen species
R-T PCR: Real-time polymerase chain reaction
SOD: Superoxide dismutase
TGF-β: Transforming growth factor-β
TNF-α: Tumor necrosis factor alpha
